# The potential of a novel enzyme-based surface plasmon resonance biosensor for direct detection of dopamine

**DOI:** 10.1038/s41598-024-64796-w

**Published:** 2024-06-21

**Authors:** Safoura Jabbari, Bahareh Dabirmanesh, Sara Daneshjou, Khosro Khajeh

**Affiliations:** 1 Department of Biochemistry and Biophysics, Faculty of Advanced Sciences and Technology, Tehran Medical Sciences, Islamic Azad University, Tehran, Iran; 2 Department of Biochemistry, Faculty of Biological Science, Tarbiat Modares University, P.O. Box 14115-175, Tehran, Iran; 3 Department of Nanobiotechnology, Faculty of Biological Science, Tarbiat Modares University, Tehran, Iran

**Keywords:** Dopamine, Laccase, Surface plasmon resonance, Therapeutic monitoring, Biochemistry, Biological techniques

## Abstract

Dopamine is one of the significant neurotransmitters and its monitoring in biological fluids is a critical issue in healthcare and modern biomedical technology. Here, we have developed a dopamine biosensor based on surface plasmon resonance (SPR). For this purpose, the carboxymethyl dextran SPR chip was used as a surface to immobilize laccase as a bioaffinity recognition element. Data analysis exhibited that the acidic pH value is the optimal condition for dopamine interaction. Calculated kinetic affinity (K_D_) (48,545 nM), obtained from a molecular docking study, showed strong association of dopamine with the active site of laccase. The biosensor exhibited a linearity from 0.01 to 189 μg/ml and a lower detection limit of 0.1 ng/ml (signal-to-noise ratio (S/N) = 3) that is significantly higher than the most direct dopamine detecting sensors reported so far. Experiments for specificity in the presence of compounds that can co-exist with dopamine detection such as ascorbic acid, urea and l-dopa showed no significant interference. The current dopamine biosensor with high sensitivity and specificity, represent a novel detection tool that offers a label-free, simple procedure and cost effective monitoring system.

## Introduction

Dopamine is one of the significant neurotransmitters and plays vital roles in the human physiological system. Numerous diseases associated with abnormal dopamine concentration in different biological fluids^1,2^. The cardiotoxicity is accompanied by increased dopamine level and leading to rapid heart rates, hypertension, heart failure, and drug addiction. However, some other diseases such as Parkinson’s disease, schizophrenia, Alzheimer’s disease, stress and depression may cause by a low dopamine level^3^. Due to the dopamine critical and important function in the human physiological system, different considerable efforts have been invested to develop a novel analytical method for highly sensitive, selective and direct detection of dopamine to improve the quality of life and human health. Over the years, several analytical techniques including ultrahigh performance liquid chromatography-electrospray ionization-tandem mass spectrometric, capillary electrophoresis, HPLC–MS/MS, fluorescence and amperometry have been demonstrated for detection of dopamine^4–7^. All the above-mentioned techniques have their own disadvantageous and have not been considered as a real time, simple, and official technique to direct determination of dopamine. Therefore, there is a continuing challenging interest in the development of new methodology to increase sensitivity and selectivity detection of dopamine, without using time-consuming process for preparation of samples, simple procedure and with label-free detection capability. For this purpose, surface plasmon resonance (SPR) biosensing method is introduced as one of the most powerful approach for monitoring of affinity binding of biomolecules, and primary screening of druggable molecules. This analytical technique monitors the refractive index changes based on biomolecular interaction with good sensitivity that occurring at a thin metal surface (gold or silver), act exactly as a real time operating system, with no need to label or complex procedures for sample preparation^8–12^. This novel method can be utilized for therapeutic detection of dopamine in medical and clinical application.

One of the SPR strategy for detecting of analytes is based on using enzymes, because enzymes are the most widely studied system as recognition element for certain sensor applications. Some enzyme-based SPR technique are summarized in Table 1.

**Table 1:** SPR Enzyme-based biosensors.

Enzyme	Sample	Strategy	LOD	Strategy	Reference
Cholinesterase	Mycotoxin (type AFB1)	The inhibition of AFB1 on various species of cholinesterase by in vitro mutagenesis	3 μM	Enzyme-based SPR	^13^
Acetylcholinesterase	Mycotoxin (type AFB1)	Kinetic approach of aflatoxin B1-acetylcholinesterase interaction via SPR sensor	0.008 μ	Enzyme-based SPR	^14^
Thermolysin	Mycotoxin (type OTA)	The entrapment of thermolysin (TlN) into a polyvinyl alcohol/polyethylenimine (PVA/PET) polymer matrix including AuNPs and cross-linked at the surface of Au	1 nM	Enzyme-based SPR	^15^
Laccase	Chlorophene	the immobilization of laccase enzymes for their use as a receptor in the detection of chlorophene	3.72 mM	Enzyme-based SPR	^16^
Rasamsonia emersonii d-amino acid oxidase (ReDAAO)	d-amino acids	The immobilization of ReDAAO on the graphene oxide and gold nanorods composites (GO-AuNRs) design could distinguish two amino acids isomers at the same concentration	–	Enzyme-based SPR	^17^

**Figure 1:**
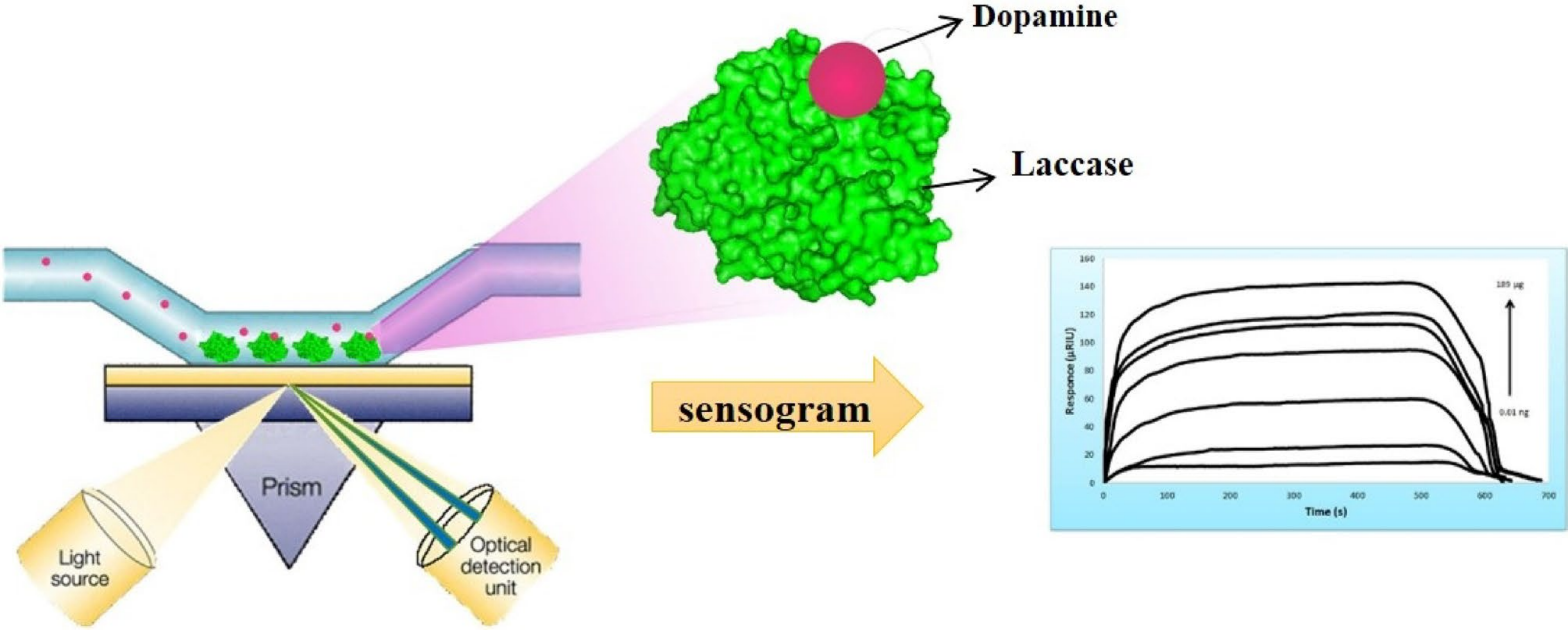
Schematic diagram of the enzyme-based surface plasmon resonance biosensor for direct detection of dopamine.

In the current study, laccase (E.C.1.10.3.2) as a multicopper-oxidase enzymes^18^ that have several industrial, biotechnological, and biocatalysis applications, was used for constructing a bioaffinity SPR based sensor to detect dopamine directly (Fig. 1). For this purpose, we have immobilized laccase on SPR-CMD chip by amine coupling procedure^19,20^. Mainly, one of the difficulties and inefficiencies in biosensor design is the loss of biomolecule activity after immobilization on the probe surface^21–23^. Here, the new biosensor demonstrated minimum loss of immobilized enzyme activity. In addition, the specificity of the immobilized laccase on the SPR chip was enhanced, towards its phenolic substrate, such as dopamine. These results, were previously reported for the first time^24^, to constructing an effective catalytic detection system in clinical applications, and could be used for cost effective and direct detection of dopamine design system. As dopamine interaction with laccase is reversible and non-covalent, the biosensor surface could be easily regenerated, using running buffer, which is mild enough that prevents severely damage the surface and allows several analysis. To determine dopamine precisely with appropriate stability and reproducibility, the experimental steps of biomolecular interactions were accurately optimized. In addition, there are always co-existing molecules such as ascorbic acid, l-Dopa and urea in organisms that interfere with the detection of dopamine. To resolve this problem, the specificity of the dopamine biosensor was assessed by testing the cross reactivity of interfering analytes such as l-dopa, ascorbic acid and urea^25–27^. The success of the present study lies in the fabrication of a new biosensor designed to directly detect dopamine. Accordingly, the proposed new method, with high specificity and sensitivity, can provide reliable evidence for the development of a SPR biosensor for therapeutic dopamine monitoring.

## Results and discussion

### Docking simulation study

AutoDock 4.2 software was used for molecular docking study to investigate the binding mode of dopamine with the laccase active site. The in silico docking demonstrated a remarkable binding and revealed that the residues involved in the dopamine interaction are far enough away from the lysine amine groups for immobilization. As previously reported, laccase oriented distribution of lysine residues, are presume to develop an enzyme biosensor with appropriate selectivity, specificity, and sensitivity, that diminish the random laccase coupling^24^. These results could be used for direct detection of dopamine. To select the best docked structure, the output of docking analysis was clustered to specify the proper conformation with optimized docking energy and determine free energy of binding (Eq. (1)). The estimation of dopamine inhibition constant (K_i_) have been demonstrated in Table 2. The molecular docking analysis predicted the negative value of free energy binding that represent a favorable interaction. The results of docking also indicate that the van der Waals interactions contribution are greater than electrostatics (Table 2), that could be related to the attachment of dopamine to the hydrophobic binding site of laccase.

1 $$ \Delta {\text{G}}_{{{\text{binding}}}} = \left[ {\Delta {\text{G}}_{{{\text{intermolecular}}}} + \Delta {\text{G}}_{{{\text{internal}}}} + \Delta {\text{G}}_{{{\text{torsional}}}} } \right] - \left[ {\Delta {\text{G}}_{{{\text{unbound}}}} } \right] $$

**Table 2:** Docking analysis of laccase with Dopamine.

Compound	Est. Free energy of binding (kcal/mol)	Est. inhibition constant, Ki (uM)	Final intermolecular energy (kcal/mol)	vdW + Hbond + desolv Energy (kcal/mol)	Electrostatic energy (kcal/mol)	RMSD (°A)
Dopamine	− 6.28	24.73	− 7.78	− 6.34	− 1.44	96.2

### Laccase immobilization on the surface of CMD chip

The EDC/NHS anime coupling procedure was successfully utilized as an appropriate immobilization method to immobilize laccase (CotA) from *Bacillus* sp. HR03 on the sensor surface of the CMD 200 D chip^28^. To obtain appropriate and evaluable resonse (refractive index changes) due to the interaction of dopamine as a low molecular weight molecule, the desired amount of enzyme was immobilized^24,29,30^.

**Figure 2:**
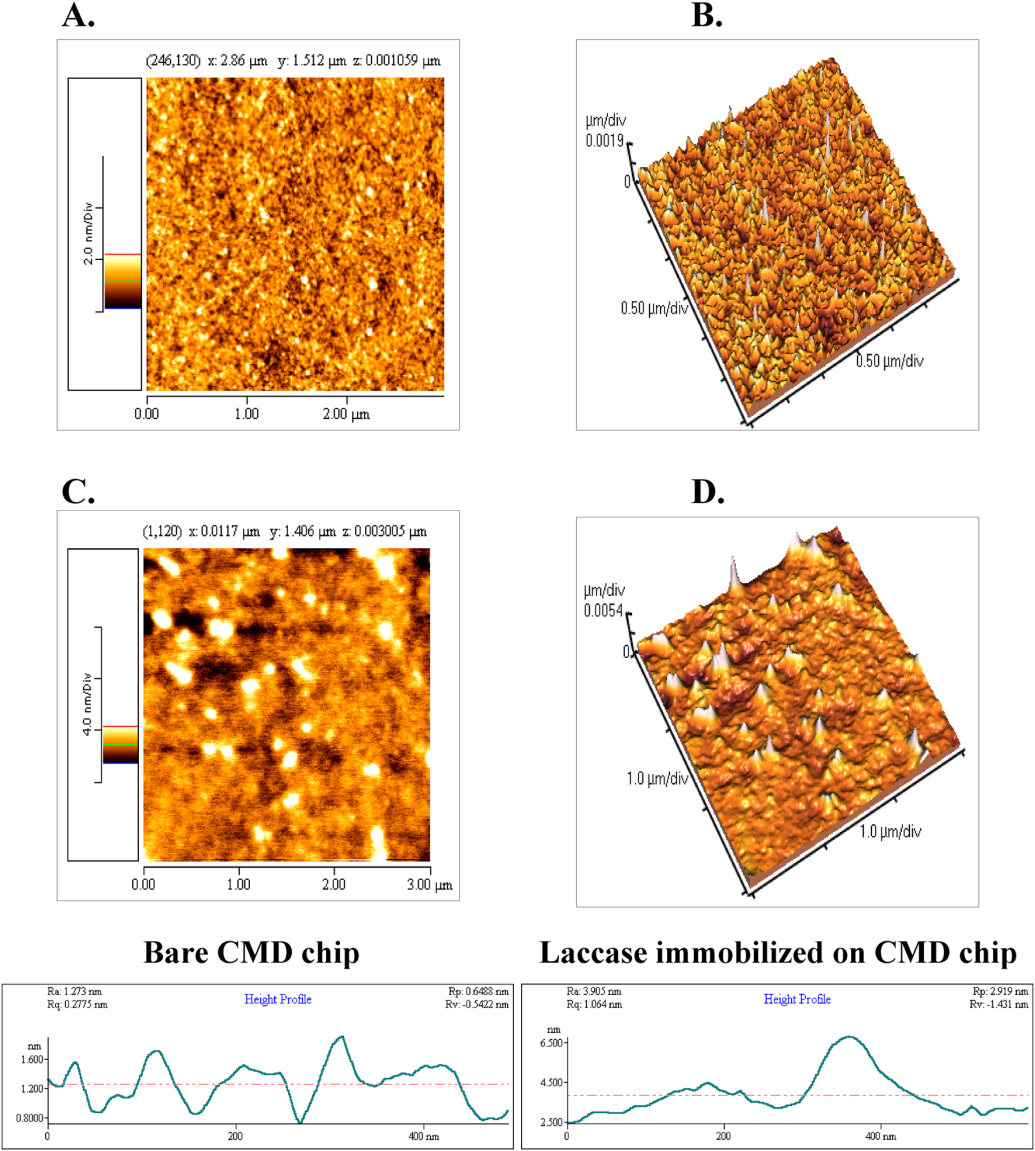
Two and three dimensional AFM micrographs of bare (**A**, **B**) and immobilized surface (**C**, **D**). Scanning area: 3 μm × 3 μm. The height profiles are shown at the bottom. The AFM images along with the height profile indicate a larger height difference on the laccase immobilized surface than on the bare CMD surface.

The sensogram of immobization is shown in the Supplementary Information (Fig. S1). In the figure S1, the AFM image of both bare and immobilized CMD chip surface revealed a homogeneous distribution of the immobilized enzyme (Fig. 2). The average surface roughness increased from 0.256 to 0.424 nm. These results were in consistent with our previous analysis^31^.

### PH optimization response and surface plasmon resonance studies

Laccase immobilization on the carboxymethyl dextran matrix was covalently performed using EDC/NHS procedure and acidic conditions (10 mm sodium acetate, pH 4.5)^24,31,32^. The pH 4.5, below the estimated pI of laccase (laccase acidic isoelectric point is around pH 5.5) was selected, because the activated carboxyl chip surface is negatively charged at pH above 4 due to the presence of carboxyl (COOH) groups. Therefore, the charge of laccase should be opposite to the surface, to electrostatically accumulate the enzyme on the surface of the sensor, which results in much higher densities of ligand immobilized.

For optimizing the pH condition to achieve the best interaction, the injected dopamine solution was continuously flowed over both the immobilized enzyme and reference surface channel, in two different pH buffer solutions (phosphate buffer pH 7.4, and pH 5.6). The reference surface channel acts as a negative control, in the SPR instrument, to correct non-specific or bulk refractive index changes caused by analyte and running buffer composition or pH^33^. The signal measured on the reference surface was systematically subtracted from the signal recorded on laccase modified surface. An increase in SPR signal after subtracting the reference signal from the sample is due to the presence of dopamine, not the pH buffer solution.

**Figure 3:**
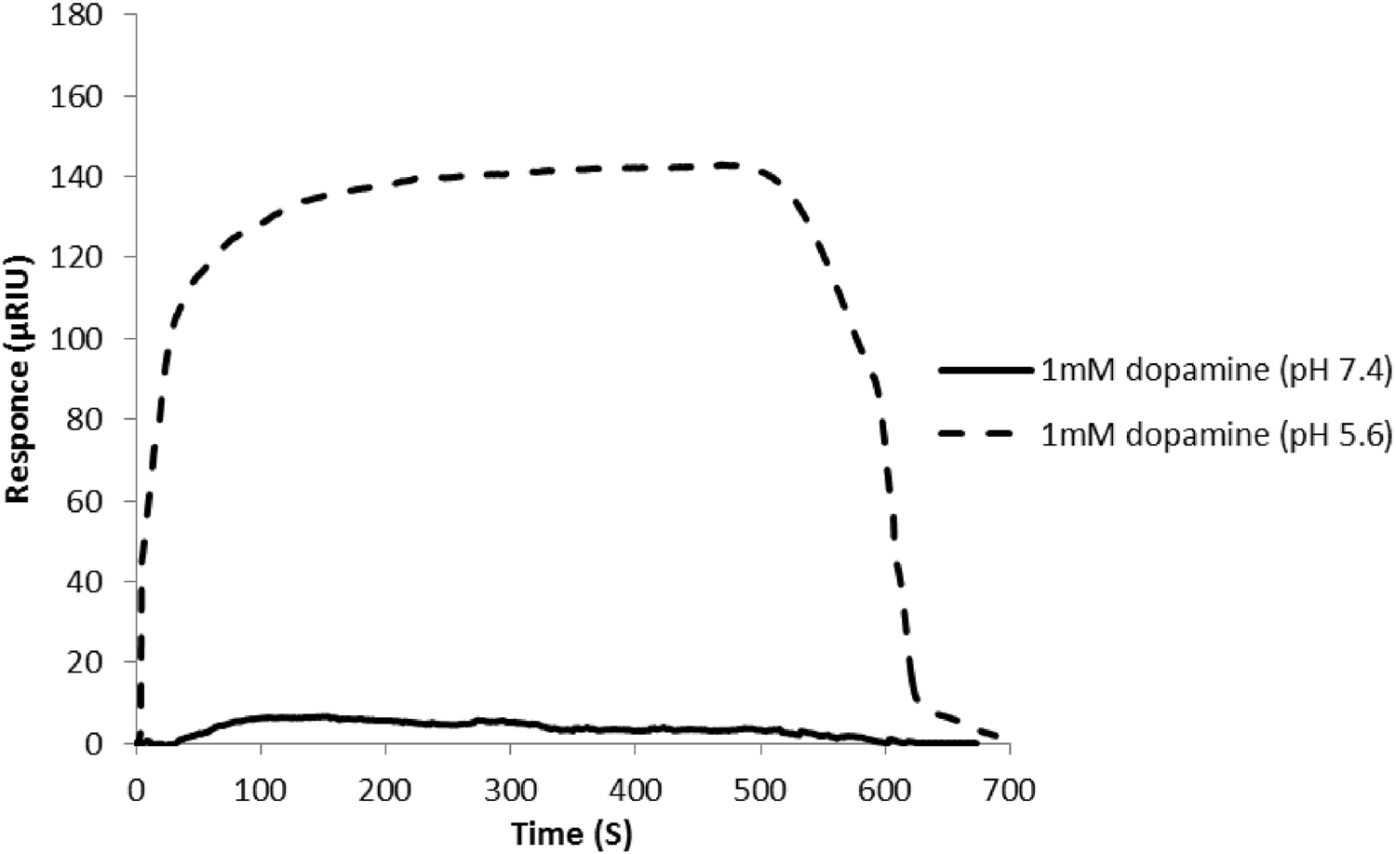
SPR response signals of 1 mM dopamine in phosphate buffer pH 7.4, and pH 5.6. Running buffer: phosphate buffer, pH 7.

Changing the environment for dopamine solutions would directly influence the interaction of dopamine and the SPR phenomenon. As the experimental data analysis indicated the obvious increase change of SPR response was in the acidic pH (Fig. 3 and Fig. S.2), therefore, the dopamine interaction was performed in pH 5.6. This shift in optimum pH of free (pH 7.4) and immobilized (pH 5.6) enzyme, could be resulted from the conformation change in immobilized enzyme and also the side chain ionization alteration of basic and acidic amino acids presents in the microenvironment surrounding the active site.

**Figure 4:**
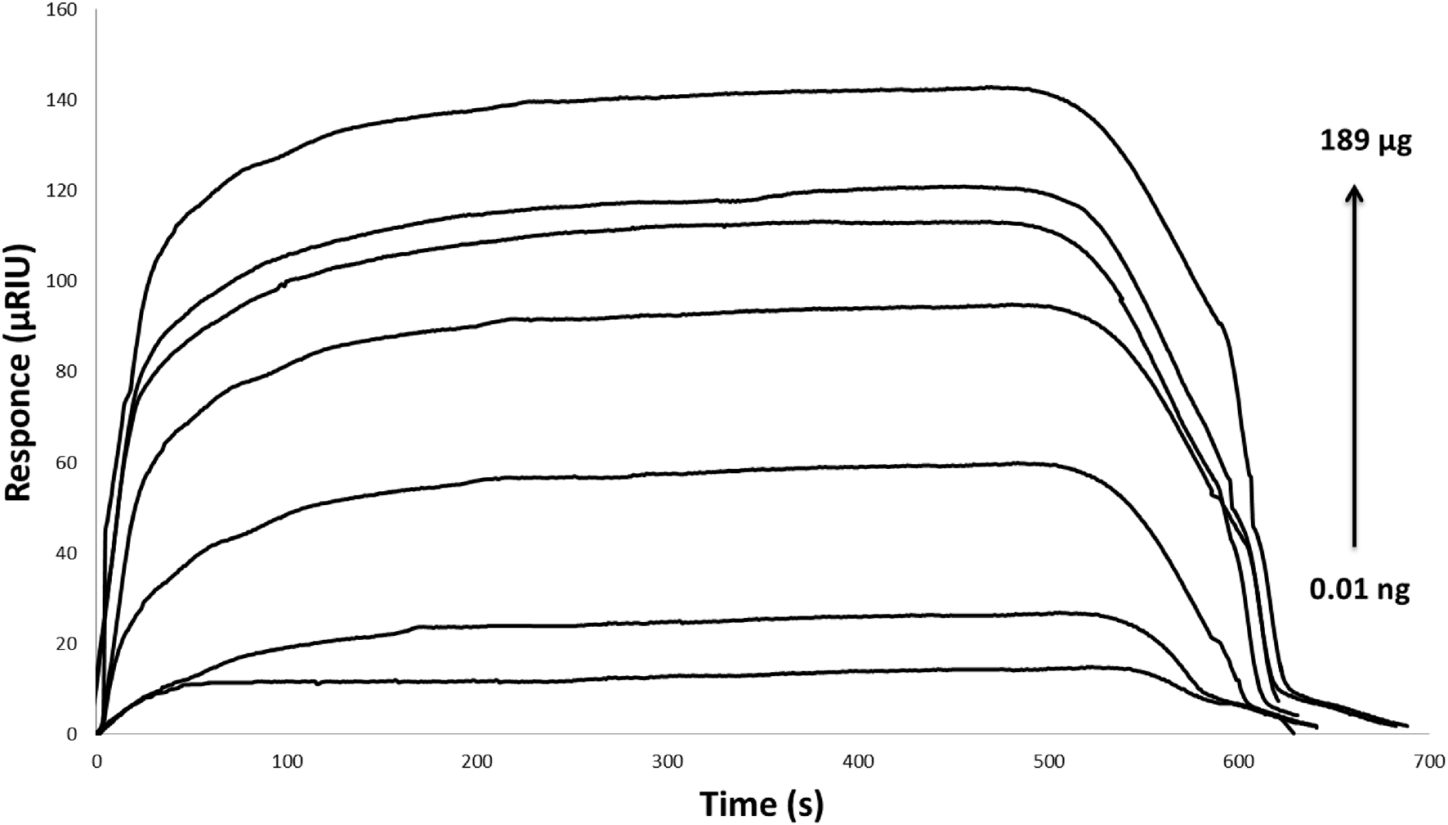
Overlay sensorgrams for the affinity reaction of laccase in the presence of 0.01 ng/ml to 189 μg/ml dopamine. Carrier solution: phosphate buffer, pH 7, flow speed: 25 μl/min, flow duration: 10 min.

In order to study the dopamine interaction with laccase, different concentrations of dopamine from 0.01 to 189 μg/ml was injected. The SPR interaction responses between dopamine and immobilized enzyme have been illustrated in Figure 4 and figure S.3. The flow of injected dopamine over the immobilized laccase displays an exclusive shape, and describes a fast and uninterrupted beginning response of injection. This monopolized pattern continues to a plateau and finally regenerates to the initial baseline (Fig. 4). Since dopamine is a small analyte, transient interaction occurs and could completely remove from the sensor chip surface by continuous flow of running buffer as regeneration solution. Although, the development of SPR biosensor for direct detection of low molecular mass analytes (between 300 and 500 Da) is a real challenge^34,35^, but here, we have demonstrated high reproducibility signals with dose–response dependency (Fig. 4).

### Determination of dissociation constant

**Figure 5:**
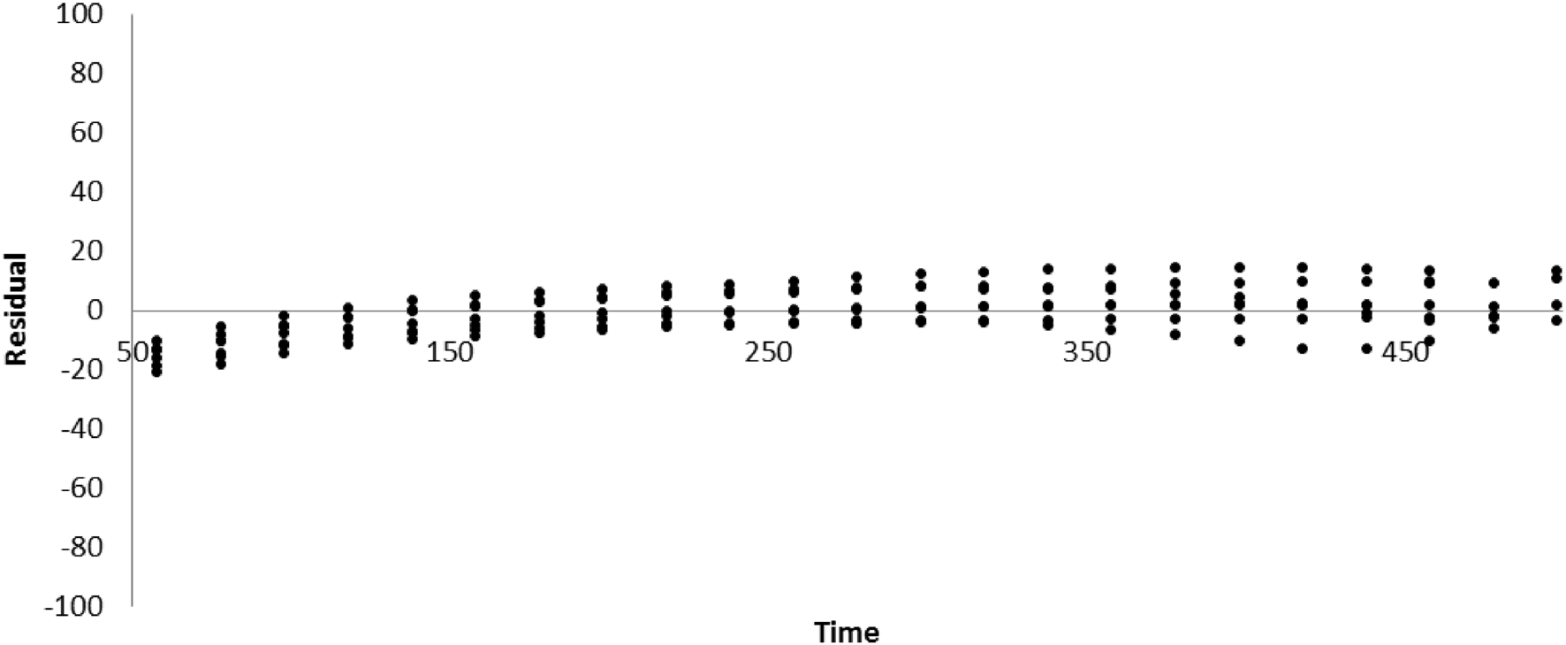
Residuals plots for Langmuir ligand model.

The recorded signals obtained from SPR was used to calculate the association and dissociation kinetic rate constants, k_on_ and k_off_, and the equilibrium dissociation constant, K_D_. To analyze the association and dissociation rate constants and the ratio of dissociation to association rate constants (K_D_) the Langmuir binding model 1:1 was used. The goodness-of-fit measures, by assessing the residual sum of squares and residual plots^36^, indicate that the particular fit is suitable. The valid X^2^ parameter and residual plot have been represented in Figure 5 and Table 3. The residuals were graphically plotted over time which revealed the residuals for 1:1 Langmuir model followed a randomly distributed (Fig. 5), indicating that this pattern acceptably describes the binding response curves.

**Table 3:** Kinetic analysis parameters.

Surface	k_a_ (M^−1^S^−1^)	k_d_ (S^−1^)	K_D_ (kinetic)	X^2a^	R_max_	Fitting model
Dopamine	1.68E + 05	8.16E−03	48.545 nM	11.871	112.7	1:1

^a^The average of the squared differences between the measured data points and the corresponding fitted values.

The mass transfer-limited binding was not influence on the kinetic diffusion parameters of dopamine small molecule, because of its high diffusion constant and fast diffusion, as well as the diffusion process have not impact on relative binding rate, to abstain from mass transport limitation^37,38^. The analysis of kinetic parameters is shown in Table 3.

The low value of K_D_ calculated from the individual k_a_ and k_d_, demonstrate the presence of an interaction with high affinity between dopamine and the immobilized enzyme^39^. Kinetic evaluation software was utilized to obtain the K_D_ and R_max_ values for calculating thermodynamic parameter and G binding, using the equations below^40^

2 $$ {\text{G}}_{{{\text{binding}}}} = - RT{\text{lnK}}_{{\text{A}}} $$

3 $$ {\text{K}}_{{\text{A}}} = {1}/{\text{K}}_{{\text{D}}} $$

where R is the universal gas constant, K_A_ is the affinity constant, T is temperature in degrees of Kelvin. G_binding_ represents the change of Gibb’s free energy of binding interaction between dopamine and immobilized laccase. The G_binding_ negative value of this interaction (− 9.5 kJ/mol or − 2.3 kcal/mol), in consistent with our new results of molecular docking analysis, confirm the correctness of spontaneous interaction between dopamine and laccase enzyme that beforehand has been immobilized on the CMD chip surface.

### Sensitivity and limit of detection

**Figure 6:**
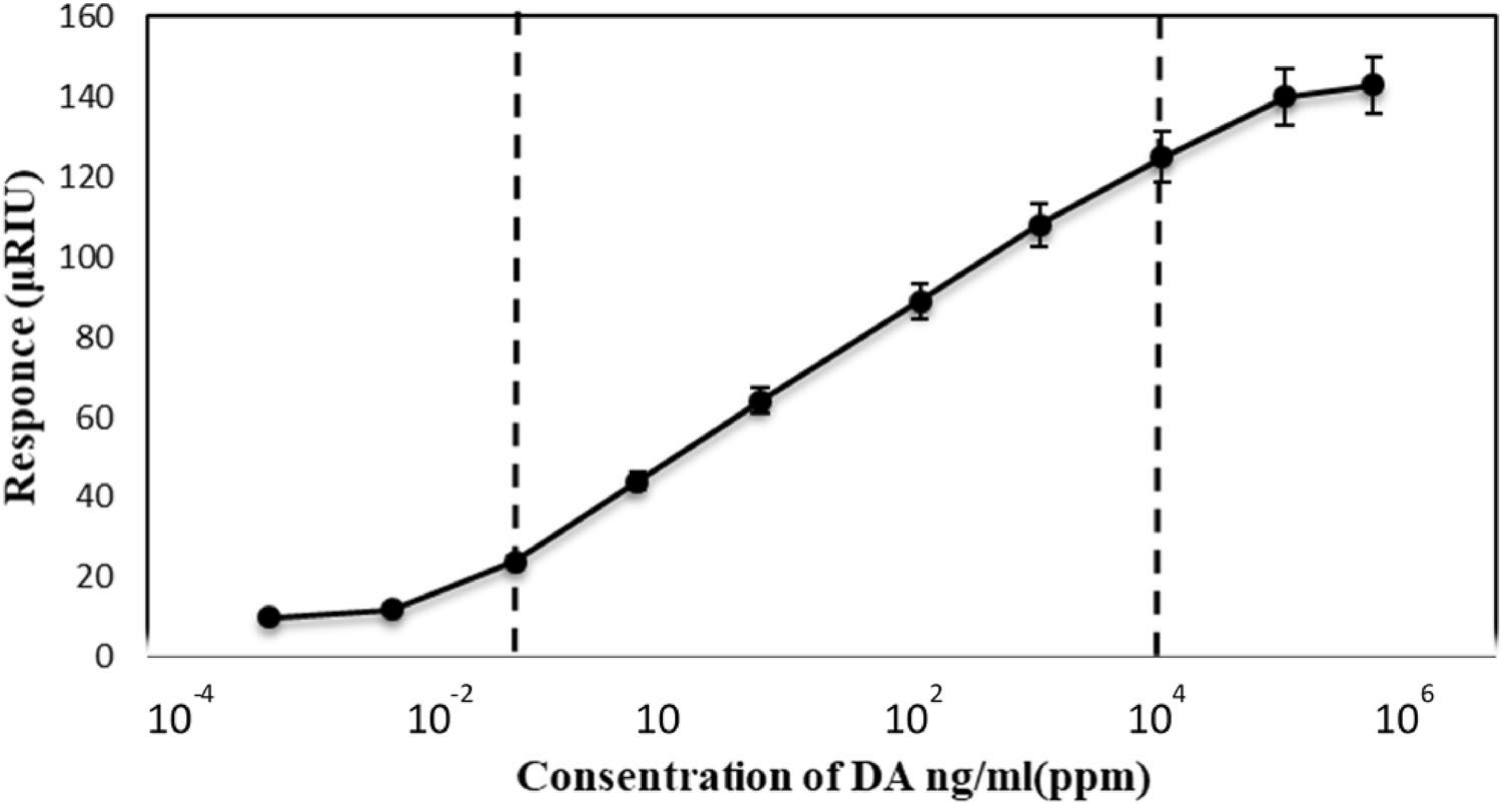
Calibration plot for the detection of dopamine using immobilization. The error bars represent the standard deviation of measurements from 3 times repetitions. The calculated linear equation is y = 20.6x–17.2.

To characterize this SPR biosensor, its response to the dopamine concentration (from 0.01 to 189 μg/ml) was investigated. Based on the dopamine standard curve (Fig. 6), an association between signal changes and dopamine levels could be detected. Under optimized experimental conditions the biosensor exhibited a linearity from 0.01 to 189 μg/ml and a lower limit of detection of 0.1 ng/ml (S/N = 3). The sensitivity was significantly higher than that of the most direct dopamine detection sensors reported to date. This result represents a remarkable clinical achievement, as normal concentrations of dopamine (between 10 and 1000 nM) in nervous and body fluids are very low. Therefore, it could be used for neural analyses.

The comparison of different enzyme-based biosensors used to detect dopamine is shown in Table 4.

**Table 4:** Enzyme-based biosensor used to detect dopamine.

Enzyme	Samples	LOD (nM)	Linear range (μM)	Technique	References
Laccase	Dopamine injection sample	5.2	0.0052–100	SPR	This study
Polyphenol oxidase	–	50	0.050–0.08	Electrochemical method	^27^
Tyramine oxidase. HRP	PC12 cell	10	0.001–1	Enzyme-catalyzed luminescence method	^41^
HRP	Dopamine injection sample	600	15–865	Voltammetric study	^42^
Laccase	Human plasma, pharmaceutical samples	29	0.5–13	Electrochemical method	^18^
Laccase	Pharmaceutical injection	180	1.5–7.5	Electrochemical Method	^43^
Tyrosinase	–	5 × 10^3^	5–50	Electrochemical method	^44^
Tyrosinase	Human urine	30	0.01–1000	Fluorescence study	^45^
Tyrosinase	Human serum	60	0.1–6.0	Fluorescence study	^46^
Tyrosinase	Serum samples	3.1 × 10^−3^	0.0075 × 10^−3^–1.5 × 10^−3^	Electrochemical method	^47^
Tyrosinase	Rat brain in vivo	1.0	0.01–220	Electrochemical method	^48^

The biosensor specificity was examined in the presence of possible coexisting compounds for dopamine detection. The response signal corresponded to the addition of 1 mg/ml ascorbic acid, l-dopa and urea and revealed no significant interference. Future details are provided in the Supplementary Information (Fig. S.4).

## Conclusion

In this study, we have represented a novel SPR biosensing strategy for direct detection of dopamine using laccase as a sensor biorecognition ligand. Dopamine is a low-molecular-weight compound thus its direct detection is difficult using SPR and has not been reported. As, the refractive index is sensitive to changes in the conformation of the immobilized protein, therefore, the binding of dopamine to laccase active site can induce detectable changes in the SPR signal.

As dopamine is the laccase substrate, the interaction could be occurring specifically. Therefore, this study suggests simple and cost-effective biosensor design, instead of using expensive antibodies for specific detection.

In addition, the use of immobilized laccase as bio-recognition elements in SPR biosensors could be preferable, since not only enzyme immobilization is a useful technology to increase the thermal and chemical stability required for many medical and biotechnological applications such as biosensors but also, bacterial laccases are usually possess high stability under drastic conditions and our results in the laboratory showed that the laccase (CotA) from *Bacillus* sp. HR0 has high-level intrinsic stability^49–51^. Moreover, the conformational changes in immobilized enzyme enhance the laccase activity toward its phenolic substrates (such as dopamine) by shifting the optimal pH to acidic value. This simple procedure makes laccase a desirable biorecognition candidate compared to dopamine receptor, which has low levels of expression, solubility challenges and time-consuming protocols. As shown in Table.4, among different enzyme-based biosensors with various LOD and linear range for dopamine detection, our study represent acceptable, remarkable and high sensitivity with a linear range from 0.1 to 18.9 μg/ml and lower detection limit of 0.1 ng/ml. This novel, label-free, real-time, fast and simple biosensing technique based on SPR and enzyme, could introduce an supportable clinical technique to monitor dopamine therapeutically. Dopamine is one of the significant neurotransmitters that its abnormal concentration is associated with CNS disorders. The present investigated strategy could provide a novel opportunity for powerful and economic CNS disorders diagnosis.

## Material and methods

### Materials

All chemicals were prepared from Sigma–Aldrich Chemical (USA). The sensor chip, carboxymethyldextran (CMD 200 M), and the amine-coupling kit comprising *N-*ethyl-*N*′-(3-diethylaminopropyl) carbodiimide (EDC), *N*-hydroxysuccinimide (NHS), and ethanolamine hydrochloride were acquired from Xantec Bioanalytics (Germany). Laccase (CotA) from *Bacillus* sp. HR03 was expressed in *E. coli* BL21 (DE3) cells in our laboratory^49^.

### Methods

#### Laccase immobilization on the surface of CMD chip

SPRSR7500DC instrument (XanTec bioanalytics GmbH, Gernmany) was used for SPR measurements with an automatic flow injection system. The immobilization protocol was performed through EDC/NHS esters, via the primary amine groups (lysine residues) of enzyme^24,52,53^. A 10 min pulse of 250 µl NHS/EDC injection was performed for activation of surface carboxyl groups, and followed by 250 μl of laccase (0.2 mg/ml) in 10 mM acetate buffer (pH 4.5) injection, to immobilize enzyme on the activated chip. The remaining esters were deactivated by 1 M ethanolamine (pH 8.5). The deactivation process could remove any remaining electrostatically bound enzyme. The procedure was followed by rinsing all of the mentioned operations with a phosphate buffer solution. The surface of the reference channel is also activated and blocked with NHS/EDC and ethanolamine respectively.

The sensogram of immobization is shown in the Supplementary Information (Fig. S1). In the figure S1, the SPR signal between 800 and 1300 s is related to 10 mM NaOH/2 M NaCl washing solution, as a pre-treatment step for removing any non-covalently bound ligand or contaminant, and also obtain baseline for amine coupling immobilization procedure. The red line shows the change in refractive index of the reference flow cell (Left channel of sensor chip), with no immobilized enzyme, blue line shows the change in refractive index of the sample flow cell (Right channel of sensor chip), with immobilized enzyme, and pink curve shows the difference between red and blue curve. In the surface activation step by NHS/EDC solution, there is no immobilization and interaction, so the pink line does not show any response. Over amine coupling process, the difference curve indicates, the signal up to 5000 μRIU, that has been achieved by laccase immobilization on the surface.

The increased SPR signal is due to a change in the refractive index and these changes measured in microrefractive-index units (μRIU). As 1 RU is equivalent to a surface concentration of 10 mg/l, the signal increasement, up to 5000 μRIU, indicates the concentration of 50 mg/l of the ligand on the sensor surface. Any mass change of interacting analyte with the surface chip could straightly change the refractive index.

#### The studies of atomic force microscopy (AFM)

AFM Instrument (Veeco-Autoprobe-CP-research) was applied to record the topology images of the CMD SPR-chip surface. The condition of AFM imaging was accomplished in non-contact mode at room temperature in air with a resolution of 256 pixels for all AFM images^31^.

#### Enzyme activity assay

As previously described^49^, to measure the activity of laccase enzyme, its two substrates, ABTS (2 mM) and SGZ, were prepared in 100 mM phosphate buffer (pH 4) and 100 mM phosphate buffer (pH 7), respectively. The ABTS oxidation was monitored by the increase in absorbance at 420 nm (= 36,000 M^−1^/cm) and SGZ at 525 nm (= 65,000 M^−1^/cm)^54,55^. One unit of enzyme catalytic activity is described as the amount of enzyme that catalyzed 1 mol of substrate per minute at 25 °C.

#### Optimizing pH condition and

After setting up the immobilized CMD chip onto the SPR instrument, the flowing of dopamine over the sensor surface was studied. For optimizing the pH condition to select the best binding interaction, two different pH (phosphate buffer 5.6 and 7.4), below dopamine isoelectric point, was invested, in the range of 6–1000 μm of dopamine.

#### SPR measurements

To achieve the good binging interaction, the pH condition was optimized by studying the phosphate buffer pH 5.6 and 7.6, followed by injection of dopamine concentration range in optimized buffer solution, including: 0.01 ng/ml, 0.1 ng/ml, 1 ng/ml, 10 ng/ml, 1.89 μgr, 18.9 μgr and 189 μgr/ml, over the sensor chip. The injected analyte solutions were continuously flowed over both the immobilized laccase and reference surface. The flow rate of the injections was 25 μl/min at 25 °C and performed in 10 min (including 120 s association time)^56^. The flow of regeneration solution (running buffer) was continued after each steps of dopamine injection to completely remove the analyte from the sensor chip surface, since the dopamine small analyte displayed transient interaction. The blank control buffer without dopamine (phosphate buffer), was injected as a negative control. The subtraction of blank injection (zero analyte concentration) can compensate for drift and small differences between the reference and active channel. The reference subtraction and blank subtraction are often referred to as double referencing^57^.

Reference sensorgrams were subtracted from binding sensorgrams using the Scrubber analysis program (Biologic Software Pty. Ltd., Canberra, Australia). The same program was used to analyze the residuals plots, Langmuir ligand model and kinetic parameters. To evaluate the specificity of the biosensor, the cross-reactivity of structural dopamine analogous was tested. The reproducibility and stability of the biosensor was also studied.

### Supplementary Information

Supplementary Information.

## Data Availability

All available data in present article has been illustrated through Figs. 1 to 6, Table 1 to 4 and Figs. S.1 to S.4.
