# Supplementary material for: The potential of a novel enzyme-based surface plasmon resonance biosensor for direct detection of dopamine
Source: Sci Rep. 2024 Jun 21;14:14303. doi: 10.1038/s41598-024-64796-w (PMC11192927; doi:10.1038/s41598-024-64796-w)
Supplement: Supplementary file 1 — Supplementary Information. [file 41598_2024_64796_MOESM1_ESM.docx]

S. 1. Sensogram for covalent immobilization of laccase onto SPR-CMD surface. Carrier solution: 20 mM phosphate buffer (pH 7), ﬂow speed 25 l/min, ﬂow duration: 10 min. Red line shows the change in refractive index of the reference flow cell (Left channel of sensor chip), with no immobilized enzyme, Blue line shows the change in refractive index of the sample flow cell (Right channel of sensor chip), with immobilized enzyme, Pink curve shows the difference between red and blue curve. The difference curve indicates, over amine coupling process, the signal up to 5000 μRIU has been achieved by laccase immobilization on the surface. As laccase should covalently immobilize on the chip surface, it cannot be removed from the sensor chip by continuous flow of running buffer, and the SPR response does not return to baseline.


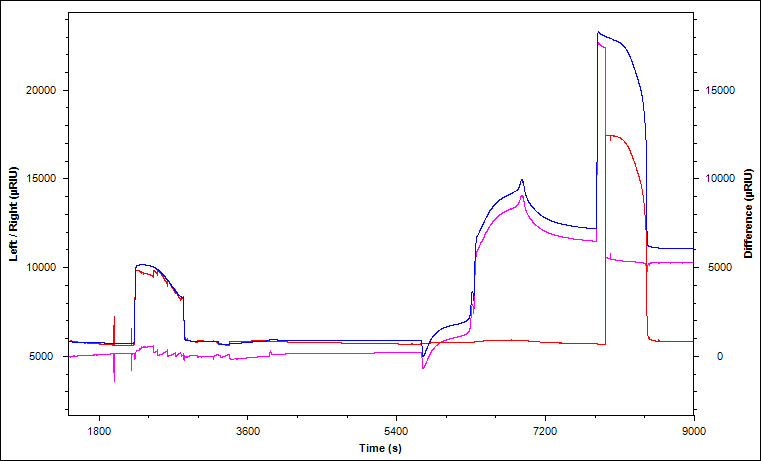


**Ethanolamine**

**(Blocking)**

**EDC/NHS**

**Laccase in 10mM**

**sodium acetate buffer**

**Immobilized Laccase**

S.2. The SPR response signals of 1mM dopamine in phosphate buffer pH 7.4, and pH 5.6. Red line shows the change in refractive index of the reference flow cell (Left channel of sensor chip), with no immobilized enzyme, Blue line shows the change in refractive index of the sample flow cell (Right channel of sensor chip), with immobilized enzyme, Pink curve shows the difference between red and blue curve. The increment of pink difference curve indicates the present of interaction between analyte and immobilized enzyme. The blue arrows show the point of dopamine injection in different pH (pH 7.4, and pH 5.6), simultaneously over the immobilized laccase and the reference surface. As it is shown, the obvious increase change of SPR response (pink curve) is in the pH 5.6 and no change in the pH 7.4, therefore the dopamine interaction was performed in pH 5.6. Since dopamine is laccase substrate and a small analyte, non-covalent transient interaction occurs and the analyte could completely remove from the sensor chip surface by continuous flow of running buffer as regeneration solution which prevent the cumulative concentration


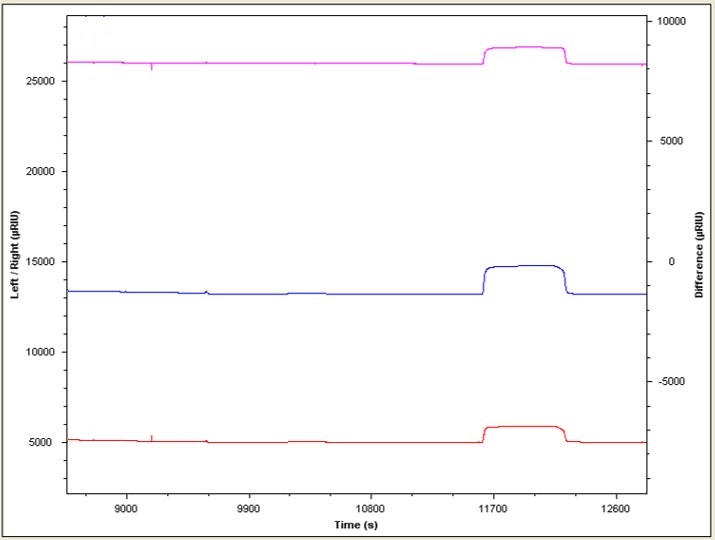


**1mM dopamine phosphate buffer**

**pH 7.4**

**1mM dopamine phosphate buffer pH 5.6**


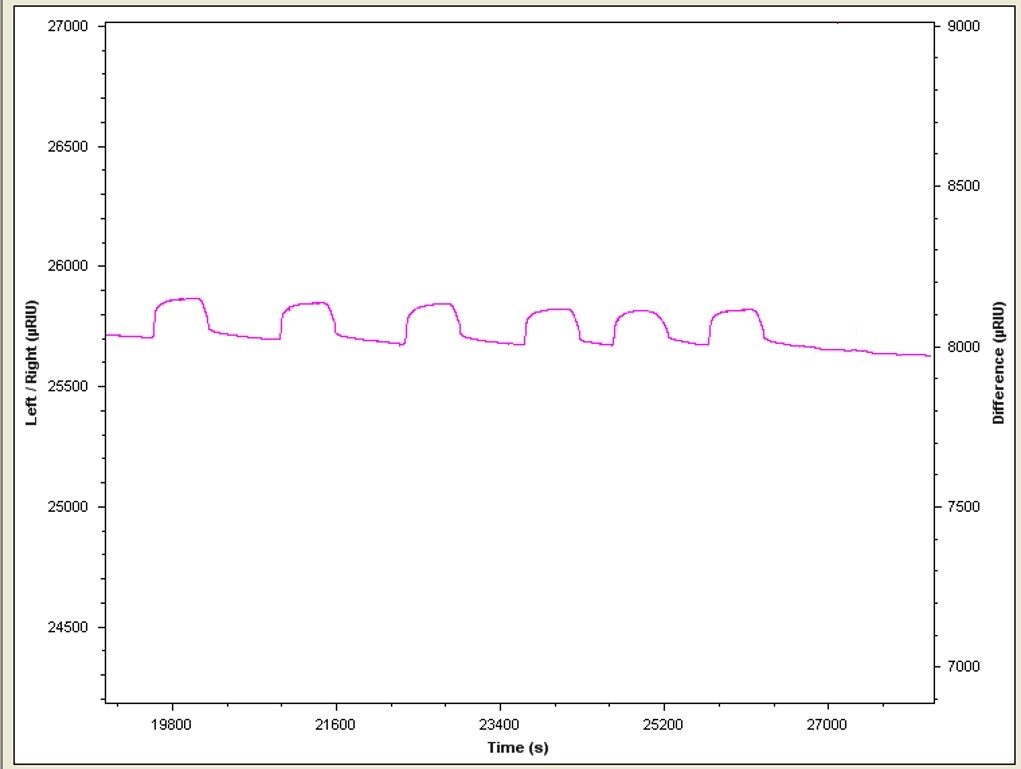
S.3. The sensogram of different dopamine injection over immobilized laccase. The real-time measurement between time 19000 s and 26000 s (nearly 2 hours) indicates the obvious increase change of SPR response during time.

S.4. The comparison of response signal in the presence of 1mg/ml Ascorbic acid, 1mg/ml Urea, 1mg/ml L-Dopa and 10 ng/ml Dopamine. Carrier solution: phosphate buffer, pH7, ﬂow speed: 25 μl/min, ﬂow duration: 10 min.


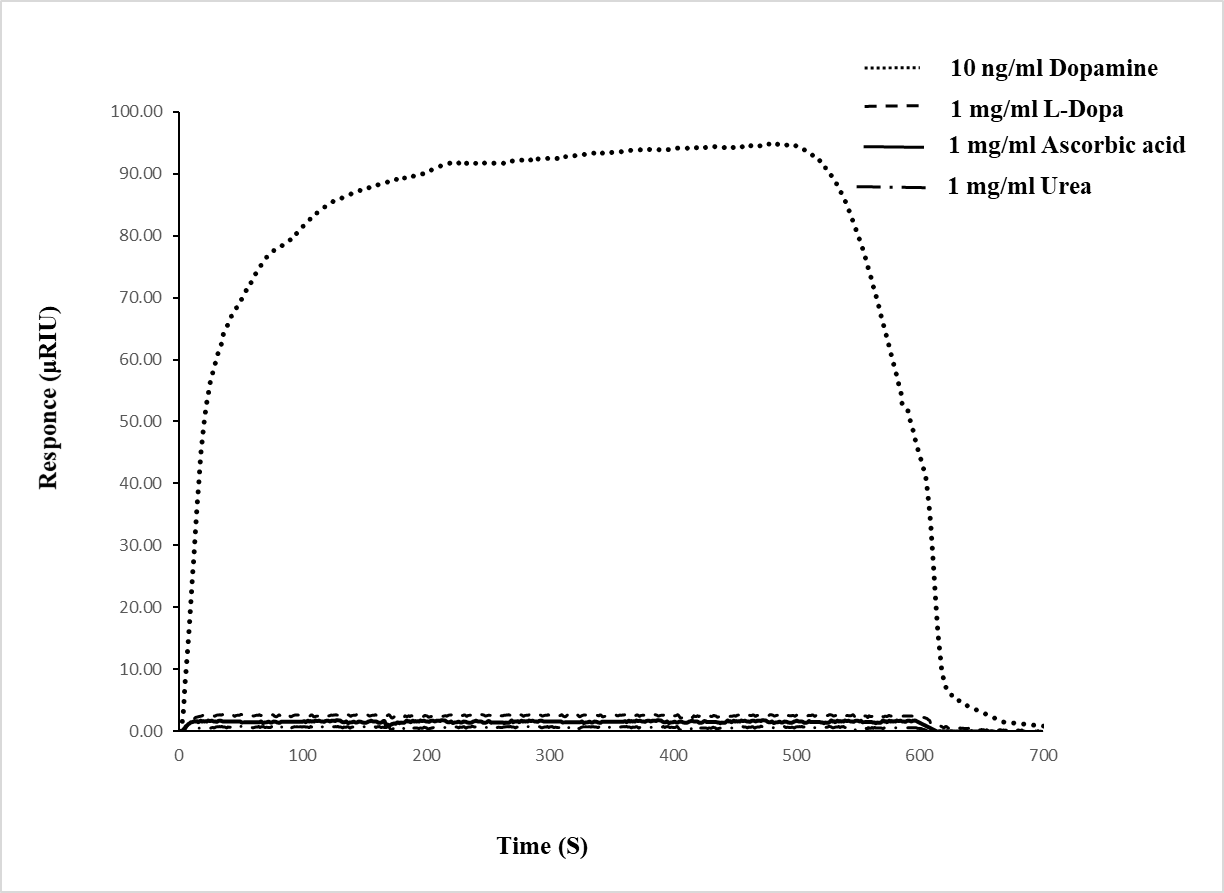


***Protein-ligand blind docking method:***

AutoDock 4.2 software was employed for docking analysis. The interaction grid box with 126 × 126 × 126 points in x, y and z directions with 0.553Å spacing, including all the binding pocket residues and the ligand structure, had been selected. 100 docking runs for Dopamine had been estimated to be enough to list the possible alternative positions of the small ligand in the little laccase binding pocket. The best result obtained in the docking simulation was analyzed.
